# Influence of semiconductor and metal nanoparticles on the dielectric properties of ionic matrix cadmium octanoate

**DOI:** 10.1186/s11671-014-0723-0

**Published:** 2015-02-13

**Authors:** Dmytro Zhulai, Denys Fedorenko, Alexander Kovalchuk, Svetlana Bugaychuk, Gertruda Vasylivna Klimusheva, Tetyana Alfredivna Mirnaya

**Affiliations:** Institute of Physics of National Academy of Sciences of Ukraine, Nauky prosp., 46, Kyiv, 03028 Ukraine; National University of Technologies and Design, Nemirovich-Danchenko 2, Kyiv, 01011 Ukraine; V. I. Vernadsky Institute of General and Inorganic Chemistry of National Academy of Sciences of Ukraine, Academician Paladin prosp., 32-34, Kyiv, 03142 Ukraine

**Keywords:** Ionic liquid crystal, Nanocomposite, Dielectric conductivity, CdS, Au quantum dots

## Abstract

Dielectric properties of ionic composites consisted of cadmium octanoate matrix and semiconductor or metal nanoparticles have been investigated. The nanoparticles of different nature (semiconductor CdS, metal Au, and metal core-semiconductor shell Au-CdS) were chemically synthesized in the smectic A phase of (Cd^+2^(C_7_H_15_COO)^−2^, CdC_8_) that was used as a nanoreactor. These nanocomposites are very stable and well ordered; the size and shape of the nanoparticles (NPs) are well controlled during the synthesis. The main aim of the research was to examine the influence of nanoparticles on the dielectric properties of ionic matrix, which has smectic A ordered structure. Electrical characteristics were investigated at different temperatures, which correspond to different phases of the material. The conductivity of nanocomposites has an activation nature. The electrical conductivity anisotropy confirms the structural anisotropy of the nanocomposites. The conductivity of the nanocomposite along the cation-anion layers is higher by 2 orders of magnitude than that across the cation-anion layers. Basing on the experimental data, we proposed the simple model of the charge carriage process.

## Background

Researchers pay much attention to develop composite materials, as well as nanocomposites that exhibit new functional features. Usually, addition of nanoparticles (NPs) does not change the matrix ordering but extends the features of the composed material. Thus, this is a possibility to create nanomaterials with desired characteristics.

The new class of ionic liquid crystals based on metal alkanoates possesses a number of unique properties, such as intrinsic ionic conductivity, high solvating power, and ability to form time-stable mesomorphic glasses. The mesophase of metal alkanoates can be used as a nanoreactor for chemical synthesis and stabilization of semiconductor nanoparticles [1-4]. Earlier, electrical conductivity of lyotropic and thermotropic ionic liquid crystals of different metal alkanoate was studied [5]. The high electrical conductivity was recently observed in the potassium caproate lyotropic ionic liquid crystals (LILC). By applying an electric field, the potassium cations can easily migrate along the cation-anion layers, which contain water molecules. Thus, the conductivity along the cation-anion layers appears to be higher than that in the isotropic water electrolytes [6]. The anisotropy of the electrical conductivity in the mesophase of cobalt decanoate is as high as 4 orders of magnitude due to the alignment of the thermotropic smectic A phase.

In the present work, we investigate the electrical properties of pure cadmium octanoate matrix, as well as cadmium octanoate composites with different concentration of semiconductor, metal, or metal core-semiconductor shell nanoparticles. The main goal of the investigation is to get the material with extended electrical properties and propose the applications for these nanocomposites.

## Methods

**Figure 1:**
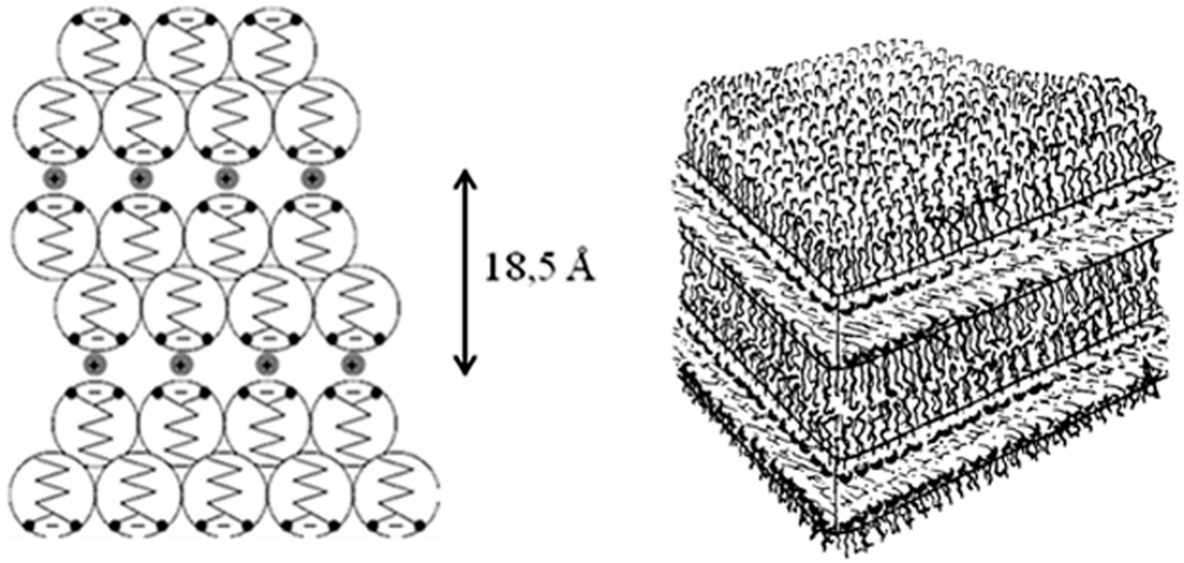
**Model of layered structure (** **smectic A)** **of cadmium octanoate CdC**_**8**_ **.**

Cadmium octanoate (Cd^+2^(C_7_H_15_COO)^−2^, CdC_8_) exists in the form of the polycrystalline powder at room temperature. Within the temperature range 98°C to 180°C, cadmium octanoate gets liquid, forming the smectic A mesophase (Figure 1), which can be used for chemical synthesis of semiconductor NPs [7]. The liquid crystalline smectic A structure of CdC_8_ can be frozen by quick cooling back to the room temperature.

We study cadmium octanoate composites containing nanoparticles of different nature. The semiconductor NPs were presented by CdS, metal Au, and metal core-semiconductor shell Au-CdS. All NPs were synthesized directly in the CdC_8_ matrix by the template-controlled method: a cadmium octanoate polycrystalline powder impregnated with a saturated aqueous-alcoholic solution of thiourea was put in a furnace at 150°C (the temperature of the mesophase of cadmium alkanoates) in argon atmosphere for 1 h. In such conditions, NPs grow in the matrix. After the synthesis, the mesophase of the composite was cooled down to room temperature. In such way, the polycrystalline powders of the nanocomposites were obtained. The presence of the NPs inside could be simply confirmed by the changes in optical absorbance spectrum. Also, the size of the NPs could be estimated by these spectra. Thus, the nanocomposites containing semiconductor CdS NPs, metal Au NPs, and metal core-semiconductor shell Au-CdS NPs were obtained.

It has been shown that the increase of the concentration of sulfide ions in the matrix does not affect the size of the NPs but increases their concentration in the matrix. Thus, the varying of the starting concentrations of thiourea allows to obtain the nanocomposites with the different concentrations of NPs. In our case, the composites with semiconductor nanoparticles used were 2, 4, and 6 mol% CdS, for the composites with metals were 2 and 4 mol% Au, and for the composites with core-semiconductor shell were 4 mol% Au + 2 mol% CdS.

X-ray measurements allowed to estimate the diameters of synthesized NPs. They appeared to be: semiconductor CdS ≈ 2.5 nm, metal Au ≈ 20 nm, and metal core-semiconductor shell Au-CdS NPs ≈ 22.5 nm. Also, the results show that the NPs have a small dispersion in their sizes; their shape is spherical.

**Figure 2:**
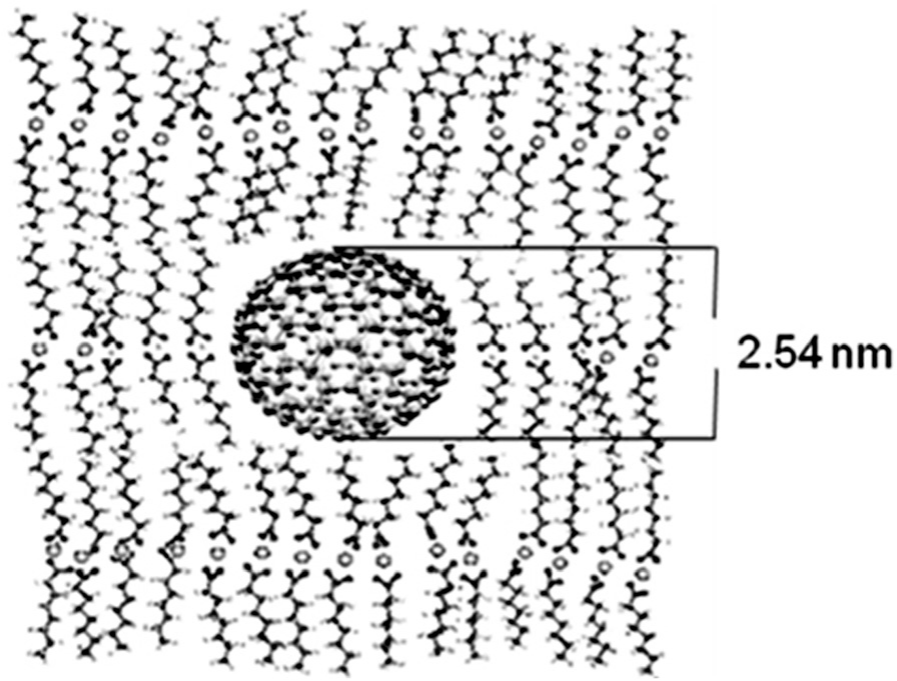
**Model of semiconductor CdS NPs in a CdC8 matrix.**

The nanocomposites are stable over a long time (years) and ordered in a layered matrix (see Figure 2) [8].

**Figure 3:**
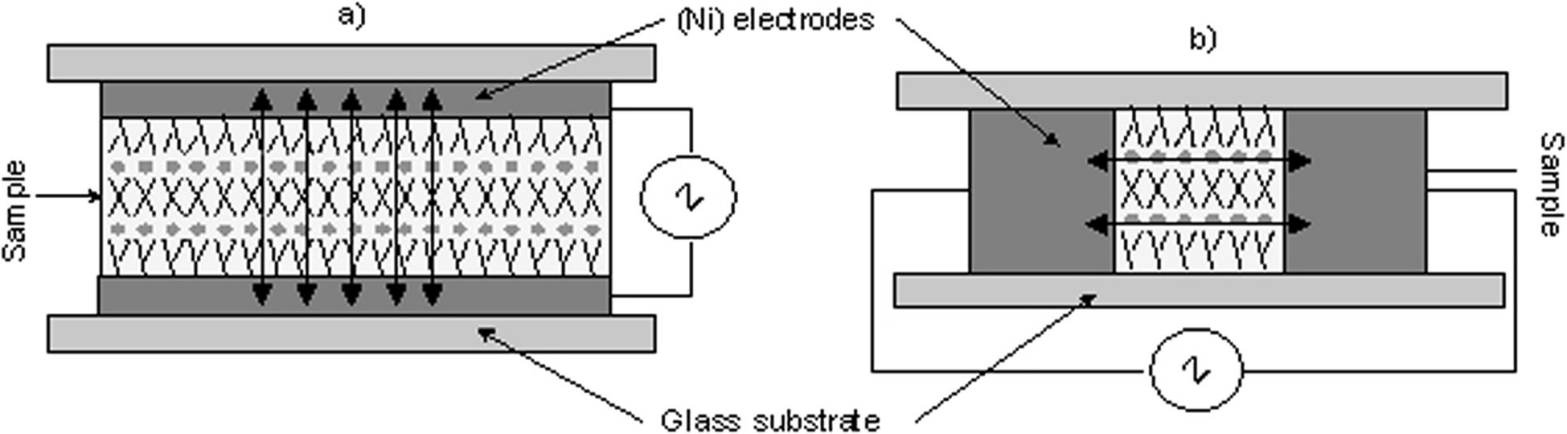
**Configurations of the cells used in experiments. (a)** Charge transfer across cation-anion layer. **(b)** Charge transfer along the cation-anion layer.

For electrical conductivity measurements, the nanocomposites were placed in flat cells with nickel electrodes and glass supports. Two geometric configurations of cells (with different location of the electrodes) were used to investigate the anisotropy of the conductivity; see Figure 3. We placed the electrodes in a plane of glass substrates to direct the electric current across (Figure 3a) the ionic layers of the nanocomposite. To direct the current along the ionic layers, the electrodes were located on the sides of the material area (Figure 3b). The spacers in the case (3a) adjusted the thicknesses of the samples. In the case (3b), the thickness is given by the thickness of the electrodes. The thicknesses of samples are chosen to be 30 μm in the case (3a) and 1 mm for the case (3b).

The electric conductivity is measured using the oscilloscopic technique [9,10]. A voltage signal has a triangular shape with the peak amplitude 0.25 V. The signal frequency was changed within the range from 50 to 10^6^ Hz. It was found that the resistance of the samples has no dispersion at frequencies above 5 × 10^3^ Hz, since the frequency 10^4^ Hz was selected to measure the electrical conductivity.

In our experiments, we measured the volumetric resistance directly. Thus, the geometric parameter, *k*, must be involved in consideration for each sample. Then, the conductivity is estimated as *σ* = *k*/*R*. The geometric parameter *k* = *l*/(*d* ⋅ *z*) depends on the sample thickness *d*, the length of the metal electrode *z*, and the distance between electrodes *l*.

To make the temperature measurements available, the samples were placed into a thermostat. The temperature inside could be adjusted in range from 20°C to 150°C, with accuracy of 1°C.

## Results and discussion

**Figure 4:**
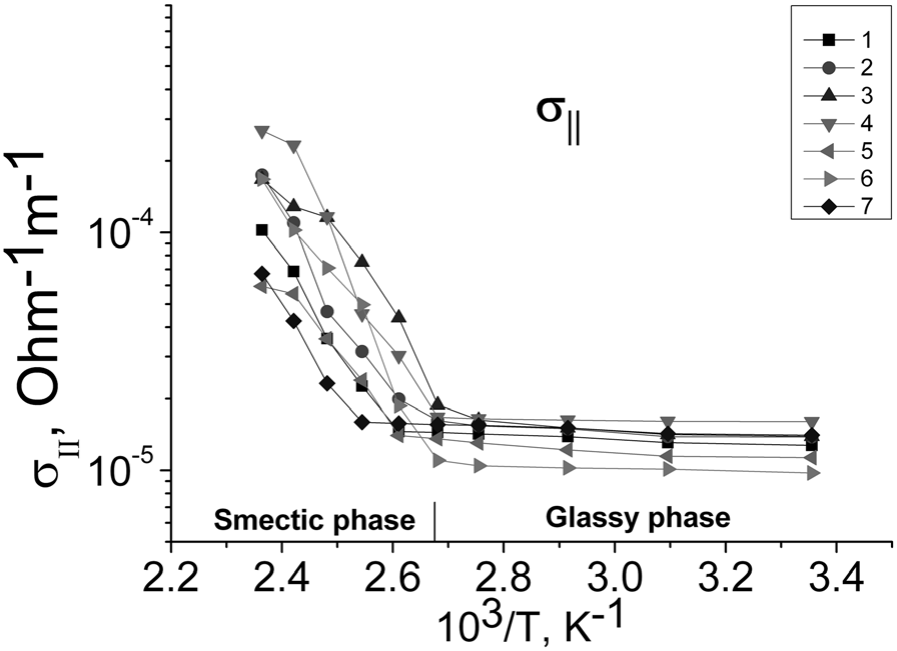
**Temperature dependence of conductivity** ***σ***_***II***_ **.** 1, CdC8; 2, CdC8 + 2% CdS; 3, CdC8 + 4% CdS; 4, CdC8 + 6% CdS; 5, CdC8 + 2% Au; 6, CdC8 + 4% Au; 7, CdC8 + 4% Au + 2% CdS; vertical line marked the phase transition temperature.

**Figure 5:**
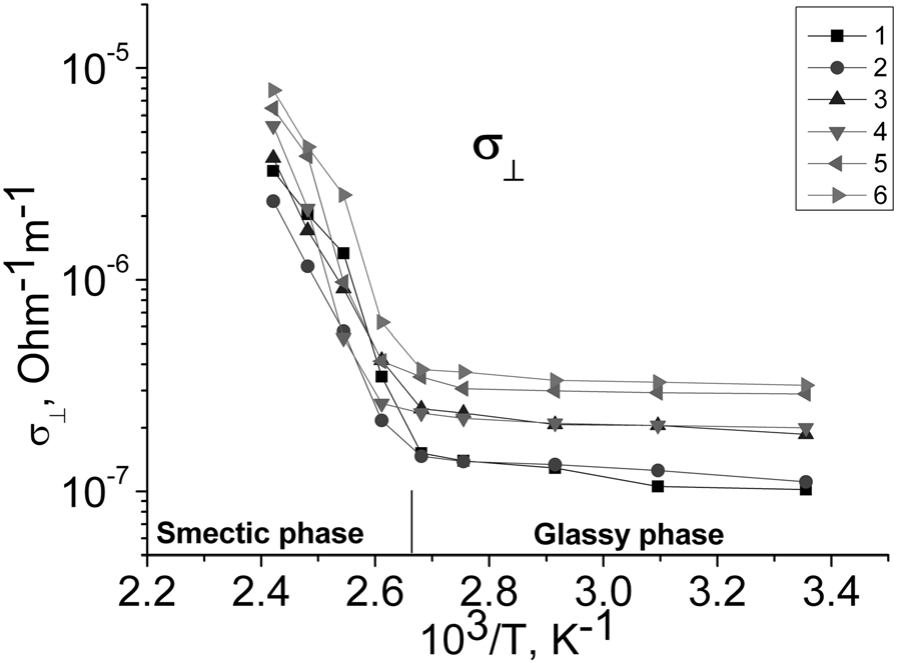
**Temperature dependence of conductivity** ***σ***_**⊥**_ **.** 1, CdC8; 2, CdC8 + 2% CdS; 3, CdC8 + 4% CdS; 4, CdC8 + 2% Au; 5, CdC8 + 4% Au; 6, CdC8 + 4% Au + 2% CdS; vertical line marked the phase transition temperature.

The electrical conductivity along cation-anion layers (*σ*_*II*_; Figure 4) and across them (*σ*_⊥_; Figure 5) was measured in samples with different concentrations of nanoparticles. We used the following concentrations: semiconductor CdS were 2, 4, and 6 mol%, metal Au 2 and 4 mol%, and metal core-semiconductor shell 4 mol% Au + 2 mol% CdS. The temperature was changed in range from a room temperature up to 150°C. The results are shown in Figure 4. It is clearly seen that the conductivity is a bit larger in the samples with the larger concentration of the NPs. Also, the conductivity along the cation-anion layers is by 2 orders of magnitude higher than that across them.

Comparison of the values of dependences *σ*_*II*_(1/*T*) at Figure 4 and *σ*_⊥_(1/*T*) at Figure 5 confirms the anisotropy of our materials. The difference in 2 orders of magnitude arises from the large difference in mobility of the charge carriers along and across of the matrix cation-anion layers. Dependencies *σ*_*II*_(1/*T*) and *σ*_⊥_(1/*T*) can be approximated by two straight lines each, since the activation energy *E*_*a*_ can be established for both phases and for each concentration of the nanoparticles using the formula (1).

At the temperatures of the mesophase existence, the dependence of *σ* (Т) can be approximated by straight lines in Arrhenius coordinates ln *σ*(1/*T*). Therefore, the conductivity of nanocomposites has an activation character and can be described by the Arrhenius formula:

1 $$ \sigma ={\sigma}_0 \exp \left(-\frac{Ea}{k{}_B{}T}\right) $$

where *σ*_0_ is the parameter, which depends on the phase of the composite, *E*_*a*_ is the activation energy of conductivity, and *k*_*B*_ is the Boltzmann constant. Thus, we can determine the activation energy of conductivity both for different nanocomposite phases and for different design of the cells.

### Dependence of the *E*_*a*_ of composites on concentration of CdS nanoparticles and Au nanoparticles at the temperature corresponding to the glassy phas

Let us consider the glassy phase first. The activation energy *E*_*a*_ decreases with the concentration of NPs in both directions of the charge transport (along and across to the cation-anion layers). The reason of the small values of the activation energy in the glassy phase points out that the main charge is transported by electrons (Table 1).

**Table 1:** **Activation energy of conductivity on the concentration NPs in glassy phase of nanocomposites along and across cation**-**anion layers**

**Concentration of NPs in samples**	**Pure CdC8**	**2%** **CdS**	**4%** **CdS**	**6%** **CdS**	**2%** **Au**	**4%** **Au**	**4%** **Au** ** + ** **2%** **CdS**
Е ║, eV	0.017	0.015	0.013	0.003	0.33	0.16	0.009
Е _┴_, eV	0.04	0.027	0.023		0.16	0.1	0.18

### Dependence of the *E*_*a*_ of composites on the concentration of CdS nanoparticles and Au nanoparticles at temperature smectic phase existence

The conductivity along the cation-anion layers significantly grows at temperatures above 100°C when the glassy nanocomposite turns to the smectic A phase. The activation energy increases with the concentration of both semiconductor and metal NPs. In samples of nanocomposites with CdS nanoparticles, *E*_*aII*_ grows from 0.78 eV for the pure matrix to 1.24 eV for the nanocomposite with 6% CdS. In samples of nanocomposites with gold NPs, *E*_*aII*_ = 0.61 eV for 2% Au NPs and *E*_*aII*_ = 0.81 eV for 4% Au NPs. This may be due to the formation of additional charge trapping centers.

In contrary with previous case, the conductivity across the cation-anion layers decreases with the concentration of the NPs. In samples of nanocomposites with CdS nanoparticles, *E*_*a* ⊥_ decreases from 1.58 eV for the pure matrix to 0.84 eV for the nanocomposite with 4% CdS. This result can be explained if we imagine that the charge that is traveling across the cation-anion layers of matrix is pushed between molecules of alkanoate chain. Then, the increase of the NPs concentration increases the number of ‘defects’ in close packed molecules of the CdC8 matrix. Thus, the mobility of the carriers gets higher with the increase of the number of areas where the close package of the alkanoate chains is deformed. Thus, the higher concentrations of NPs provide higher mobility of the cadmium cations across the cation anion layers.

For samples containing core-shell NPs of Au + CdS, *E*_*a*_ = 0.66 eV (Table 2).

**Table 2:** **Activation energy of conductivity for smectic A phase on the concentration of NPs along and across cation**-**anion layers**

	**Pure CdC8**	**2%** **CdS**	**4%** **CdS**	**6%** **CdS**	**2%** **Au**	**4%** **Au**	**4%** **Au** ** + ** **2%** **CdS**
Е ║, eV	0.78	0.8	0.93	1.24	0.61	0.81	0.66
Е _┴_, eV	1.58	1.12	0.84		1.35	1.37	1.35

### Dependence of the anisotropy of the conductivity on the concentration of CdS and Au nanoparticles

**Figure 6:**
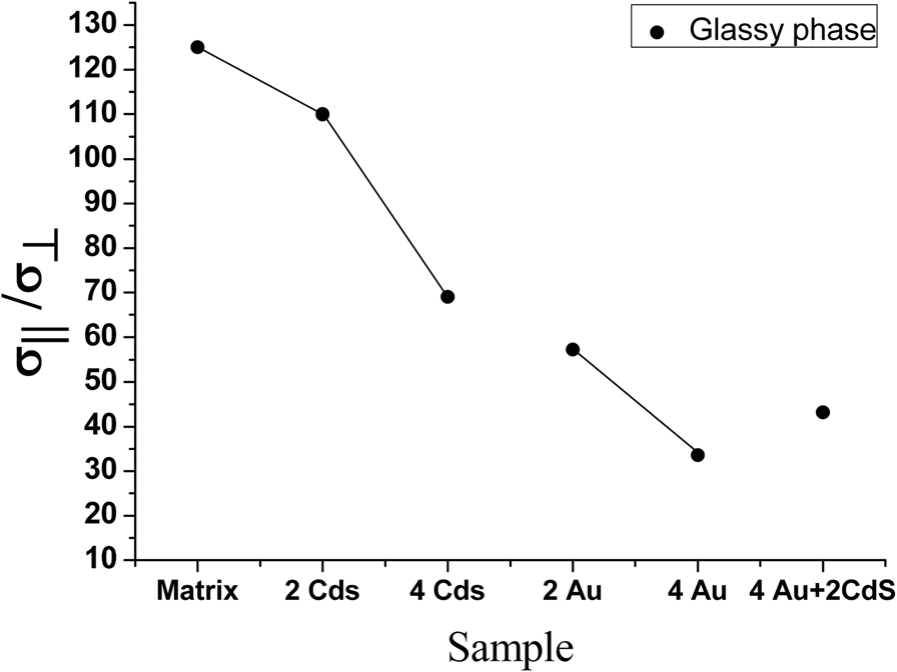
**Dependence of the anisotropy of the conductivity on the concentration of CdS and Au nanoparticles in glassy phase.**

**Figure 7:**
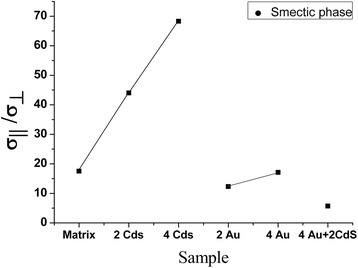
**Dependence of the anisotropy of the conductivity on the concentration of CdS and Au nanoparticles in smectic phase.**

The anisotropy of conductivity in the smectic phase of the nanocomposites is smaller than that in the glassy state. With an increase of the NPs concentration, the conductivity anisotropy increases for the smectic phase and decreases for the glassy phase. This once again proves that the CdS and Au NPs affect the ionic conductivity in both phases of the nanocomposite in a different way (Figures 6 and 7) (Table 3).

**Table 3:** **Dependence of the anisotropy of the conductivity on the concentration of CdS and Au nanoparticles**

**Concentration of NPs in samples**	**Pure CdC8**	**2% ** **CdS**	**4% ** **CdS**	**2% ** **Au**	**4%** **Au**	**4%** **Au + ** **2%** **CdS**
AG	17.5	44	68.3	12.3	17.1	5.7
AS	125	110	69	57.3	33.6	43.2

A_G_, anisotropy of the conductivity for the glassy phase; A_S_, anisotropy of the conductivity for the smectic phase.

**Figure 8:**
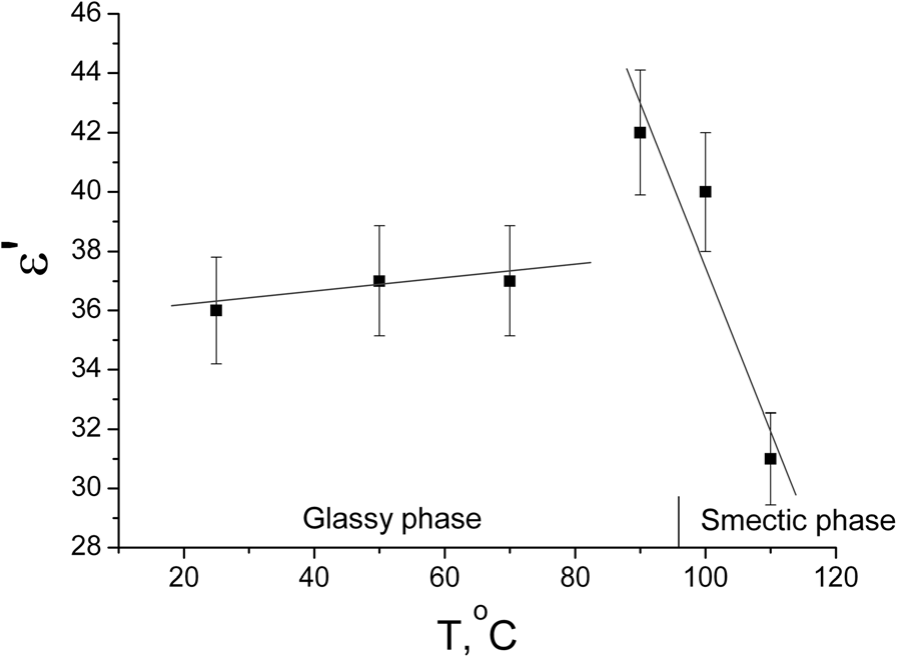
**Temperature dependence of dielectric permittivity ε**′ **of pure matrix CdC8.** Vertical line marked the phase transition temperature.

Dielectric properties of pure matrix CdC_8_ on the temperature in a range, where there is no dispersion at frequencies 20 kHz, have been studied. The frequency 20 kHz was selected to ensure the absence of dispersion. While the temperature is far from the phase transition and the dielectric permittivity, the glassy state is not depending on the temperatures. It's value is about 37 units. With the increase of the temperature up to 100°C (the phase transition from the glassy phase into the smectic phase is 98°C), we observe the increase of the dielectric permittivity up to 42 units which is very unusual. At higher temperatures, when the material completely got into the smectic phase, the dielectric permittivity decreases again, which is in a complete agreement with the theoretical predictions. The increase of the dielectric permittivity at the temperature close to the phase transition (Figure 8) needs further investigations.

## Conclusions

In general, we can conclude that the nanocomposites based on metal alkanoates with synthesized CdS, Au, and Au + CdS nanoparticles have very interesting electrical properties and are promising for applications in optoelectronics. Most important results and conclusions are the following:

- The conductivity of nanocomposites has an activating dependence in both glassy and smectic A phases.
- The conductivity of the nanocomposite along the cation-anion layers is in 2 orders of magnitude higher than across the cation-anion layers, confirming the structural anisotropy of the nanocomposite in the different phases of the materials.
- Electrons are the main charge carriers in the glassy phase. The increase of the NPs concentration brings additional free charge carriers or increases mobility of carriers.
- In the smectic phase, the increase of the nanoparticle concentration brings additional traps for the carriers, which travel in plane of the cation-anion layers. On the other hand, the nanoparticles deform the alkanoate chains and increase the mobility of the carriers traveling across the layers.
- With the increase of the concentration of CdS and Au nanoparticles, the anisotropy of the conductivity increases for the smectic phase and decreases for the glassy phase.
- The temperature dependence of the dielectric permittivity of pure matrix CdC8 is very unusual and needs to be explored more. It is found that the dielectric permittivity increases at the temperatures close to the glass-liquid crystal phase transition. Such permittivity increase is not typical for ordinary glasses and needs additional investigations.
